# Twenty Important Research Questions in Microbial Exposure and Social Equity

**DOI:** 10.1128/msystems.01240-21

**Published:** 2022-01-04

**Authors:** Jake M. Robinson, Nicole Redvers, Araceli Camargo, Christina A. Bosch, Martin F. Breed, Lisa A. Brenner, Megan A. Carney, Ashvini Chauhan, Mauna Dasari, Leslie G. Dietz, Michael Friedman, Laura Grieneisen, Andrew J. Hoisington, Patrick F. Horve, Ally Hunter, Sierra Jech, Anna Jorgensen, Christopher A. Lowry, Ioana Man, Gwynne Mhuireach, Edauri Navarro-Pérez, Euan G. Ritchie, Justin D. Stewart, Harry Watkins, Philip Weinstein, Suzanne L. Ishaq

**Affiliations:** a University of Sheffield, Department of Landscape Architecture, Sheffield, United Kingdom; b Department of Family & Community Medicine, University of North Dakota School of Medicine & Health Sciences, Grand Forks, North Dakota, USA; c Centric Lab, London, United Kingdom; d Department of Literacy, Early, Bilingual and Special Education, Kremen School of Education and Human Development, California State University, Fresno, California, USA; e College of Science and Engineering, Flinders University, Bedford Park, SA, Australia; f University of Colorado, Anschutz Medical Campus, Aurora, Colorado, USA; g School of the Environment, Florida Agricultural and Mechanical University, Tallahassee, Florida, USA; h University of Arizona, School of Anthropology and Center for Regional Food Studies, Tucson, Arizona, USA; i Department of Biological Sciences, University of Notre Dame, Notre Dame, Indiana, USA; j University of Oregon, Biology and the Built Environment Center, Eugene, Oregon, USA; k American International College of Arts and Sciences of Antigua, Antigua and Barbuda, West Indies; l Department of Genetics, Cell, and Development, University of Minnesota, Minneapolis, Minnesota, USA; m Department of System & Engineering Management, Dayton, Ohio, USA; n University of Oregon, Institute of Molecular Biology, Eugene, Oregon, USA; o Department of Student Development, University of Massachusetts, Amherst, Massachusetts, USA; p University of Colorado Boulder, Department of Ecology and Evolutionary Biology, Boulder, Colorado, USA; q Department of Landscape Architecture, University of Sheffield, Sheffield, United Kingdom; r Department of Integrative Physiology, Center for Neuroscience, and Center for Microbial Exploration, University of Colorado Boulder, Boulder, Colorado, USA; s Architectural Association School of Architecture, London, United Kingdom; t Department of Architecture, University of Oregon, Eugene, Oregon, USA; u Program of Environmental Life Sciences, School of Life Sciences, Arizona State University, Tempe, Arizona, USA; v School of Life and Environmental Sciences and Centre for Integrative Ecology, Deakin University, Burwood, VIC, Australia; w Department of Ecological Science, Faculty of Earth and Life Sciences, Vrije Universiteit Amsterdam, Amsterdam, The Netherlands; x St. Andrews Botanic Garden, Canongate, St. Andrews, Fife, United Kingdom; y Bio-integrated Design Lab, Bartlett School of Architecture, London, United Kingdom; z School of Public Health, The University of Adelaide, Adelaide, SA, Australia; aa University of Maine, School of Food and Agriculture, Orono, Maine, USA; University of California San Diego

**Keywords:** microbiomes, biopolitics, health disparities, social determinants of health, structural determinants of health, integrated research, structural determinants

## Abstract

Social and political policy, human activities, and environmental change affect the ways in which microbial communities assemble and interact with people. These factors determine how different social groups are exposed to beneficial and/or harmful microorganisms, meaning microbial exposure has an important socioecological justice context. Therefore, greater consideration of microbial exposure and social equity in research, planning, and policy is imperative. Here, we identify 20 research questions considered fundamentally important to promoting equitable exposure to beneficial microorganisms, along with safeguarding resilient societies and ecosystems. The 20 research questions we identified span seven broad themes, including the following: (i) sociocultural interactions; (ii) Indigenous community health and well-being; (iii) humans, urban ecosystems, and environmental processes; (iv) human psychology and mental health; (v) microbiomes and infectious diseases; (vi) human health and food security; and (vii) microbiome-related planning, policy, and outreach. Our goal was to summarize this growing field and to stimulate impactful research avenues while providing focus for funders and policymakers.

## INTRODUCTION

**FIG 1:**
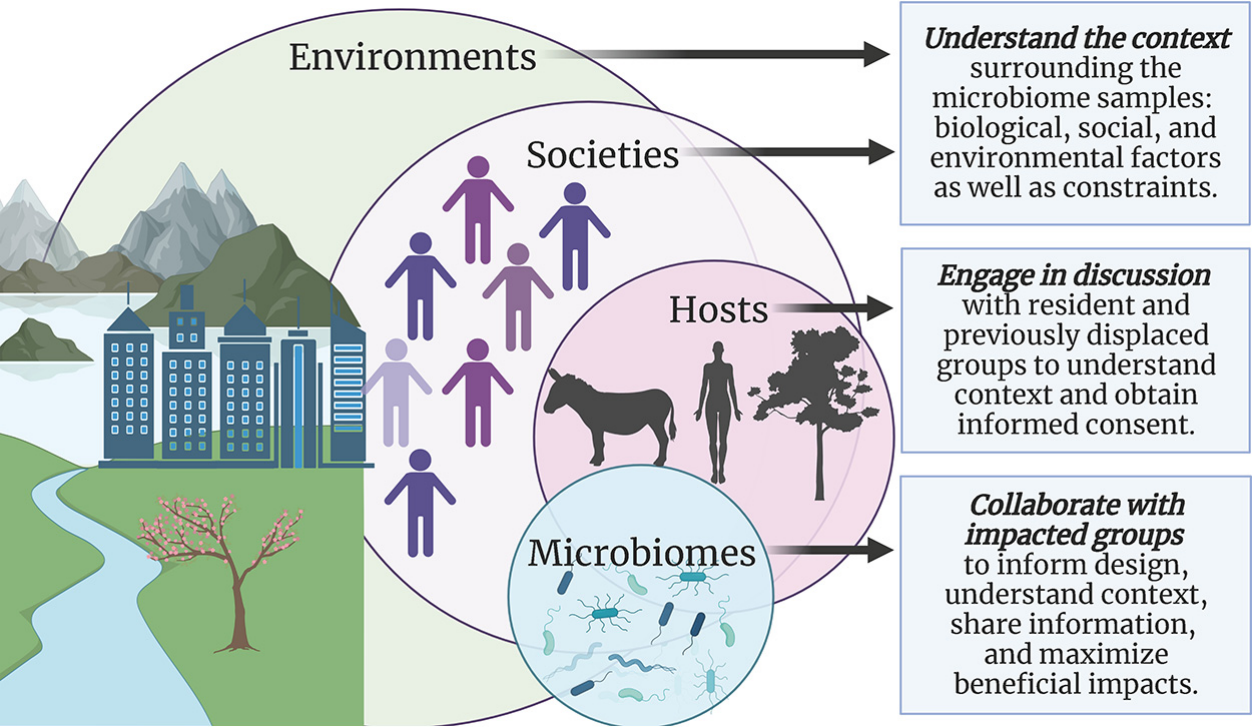
A systems-level view of microbiomes, hosts, societies, and ecosystems, and strategies to meld social equity with biology, ecology, politics, or design (made with biorender).

Rapid advances in DNA sequencing and bioinformatics have dramatically increased our ability to study the function, assembly, and complexity of microbial communities. This explosion in microbial research has revealed important insights into how microorganisms influence the functionality and resilience of ecosystems, along with human and nonhuman health. Indeed, microorganisms, including bacteria, archaea, algae, fungi, and protozoans, along with viruses, have key roles in maintaining favorable human health, but they are also fundamental to many diseases. Microorganisms are foundational to our ecosystems and contribute toward the provisioning and regulating “services” which we depend upon for survival. Moreover, recent work suggests that we need to consider microbial components of social equity and ecological (in)justice. Formed in 2020, the Microbes and Social Equity (MSE) working group collaborates on research, curricula, policy, and practice related to microbiomes and our interactions with them (Fig. 1) (1, 2).

With an overwhelming number of potential research avenues and agendas in this emerging field, it is prudent to identify important, impactful research questions and priorities to enable timely and considerate progress in the field. In this article, we present the results of an international workshop hosted by the MSE working group in late 2020. The workshop participants used an established discussion and voting-based research method to identify 20 important research questions in microbial exposure and social equity (3, 4). Similar workshops have been carried out in microbial ecology (3), conservation biology (4), and sustainability (5) and are directly applicable to the development of research and socioecological policy.

## METHODS

### Participants.

**FIG 2:**
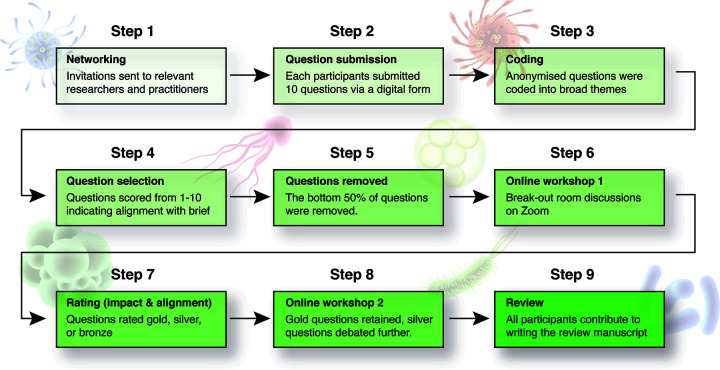
Summary of methods and workflow.

Our methods were based on those of Antwis et al. (3), which were inspired by those of Sutherland et al. (4) (Fig. 2). In brief, the MSE working group held a virtual workshop on the Zoom virtual conferencing platform in October 2020 and again in November 2020. Invitations to participate were distributed to all members of the MSE working group by e-mail. To increase diversity and rigor, all members were requested to invite one or two other experts from across different disciplines to contribute to the paper. Representing both research and practice, different nationalities, cultural groups (including Indigenous scholars), and genders, a total of 22 participants from 18 institutions around the globe made important contributions to the development of the 20 questions presented in this paper. All contributors are listed as authors.

### Questions and themes.

Prior to the two virtual workshops, all participants were asked to submit approximately 10 questions—via a digital form—that they thought most closely aligned with the following paraphrased brief from Antwis et al. (page 2 of reference 3):

“We are aiming to identify 20 questions that, if answered, will make a considerable difference to the research area of Microbes and Social Equity. These should be questions that are unanswered, could be answered, and could be tackled by a research programme. This is expected to set the agenda for future research in the area of Microbes and Social Equity”.

Participants were asked to anonymize their questions throughout the workshop stages, as well as to diversify the themes of the questions, aiming for 10 questions in total: 50% from within their discipline and 50% outside their discipline. A total of 170 questions were submitted by participants, duplicate questions were removed, and questions were subsequently coded into the following broad themes:

- Sociocultural interactions
- Indigenous community health and well-being
- Humans, urban ecosystems, and environmental processes
- Human psychology and mental health
- The microbiome and infectious diseases
- Human health and food security
- Microbiome-related planning, policy, and outreach

### Question selection process.

Prior to the first workshop, all participants were asked to score the questions from 1 to 10, whereby 1 indicated the lowest level of alignment with the brief, and 10 indicated the highest alignment. Within each theme, the top **∼**50% of questions that were most closely aligned with the brief were carried through to the first of two workshops.

To facilitate parallel sessions, we used the “breakout” room function in Zoom. These breakout rooms corresponded to the themes identified earlier in the selection process. Participants were asked to indicate which breakout room/themes they would prefer to be assigned to in advance. Questions were presented in a shared Google Sheets document, allowing participants to discuss each question remotely. Participants were asked to assign each question a gold, silver, or bronze rating depending on their potential impact, alignment with the brief, and whether the related body of literature was sufficient to support implementation (Fig. 1) or whether large knowledge gaps persisted. All of the bronze-rated questions were removed in workshop 1.

During the second workshop, we discussed which of the remaining gold- and silver-rated questions to retain. At this point, silver-rated questions could be upgraded and gold-rated questions could be downgraded. A thorough and valid justification for any upgrades or downgrades was provided and agreed upon, as required. To ensure a democratic process was upheld, participants were asked to confirm support or otherwise for any decision prior to finalizing the questions. The 20 questions included in this paper are listed below, including context and future directions, each led by one to several experts in the field (see Author Contributions) and reviewed by all authors.

## THEMES AND QUESTIONS

**Sociocultural interactions**

1. How do the microbiomes of displaced or migratory refugee populations compare to less mobile populations?
2. Are there lifetime microbial consequences of early life a dversity?
3. Given the benefits of microbial diversity, what are the effects of social isolation and institutional confinement on the health provision of microbiomes to people?

**Indigenous community health and well-being**

4. What is the relationship between speaking an Indigenous language and practicing culture to microbiome integrity?
5. What is the effect of cultural genocide on the microbiome?
6. How do research institutions learn to better engage with Indigenous and other marginalized communities in microbiome research?

**Humans, urban ecosystems, and environmental processes**

7. How does pollution affect the human microbiome and are the impacts unequally distributed?
8. How will climate change and related ecosystem degradation affect the environmental microbiome, and what will the impacts be on health?
9. How do we define microbial inequality in urban environments, and can we better identify, map, and characterize these zones?
10. How can safe and health-promoting environmental microbiomes be designed and/or restored in urban habitats to ensure equal benefits across society?

**Human psychology and mental health**

11. Is there a relationship between the microbiome and mental health, and are vulnerable populations differentially affected?

**The microbiome and infectious diseases**

12. How might infectious diseases affect human microbiomes, and does socioeconomic status influence this relationship?
13. In populations without the resources to recruit a beneficial microbiome, how can we incorporate microbiome interventions into the prevention and treatment of infections?
14. Are some communities disproportionately exposed to infectious disease agents due to anthropogenic change, such as habitat destruction, human population migration, and climate change?
15. What lessons have we learned about remote connectivity during pandemics, and what are the implications for equity and protection from infection?

**Human health and food security**

16. How do we reorganize food systems to improve food availability, security, supply chain operations, and economic viability of agriculture, while promoting access to fresh foods to support a diverse microbiome?

**Microbiome-related planning, policy, and outreach**

17. Before seeking new solutions, what are the existing community initiatives and grassroots movements that push for impacts on microbial, social, and biological equity?
18. What types of reforms are needed in healthcare and policy, in order to address our understanding of chronic, socially mediated stress on the microbiome?
19. How do we improve the translation of microbiome research findings to address social equity?
20. What is the socioecological and political potential of learning about social inequities in microbial exposure?

A review for each of the “20 important research questions in microbial exposure and social equity” is presented below under the corresponding broad themes. Each review was written by at least one expert in the respective field. Feedback was provided by the group prior to finalizing each review.

## I. SOCIOCULTURAL INTERACTIONS

An individual’s early life experiences can have profound impacts on their lifetime health. Moreover, sociocultural interactions play key roles in shaping the disparities in health outcomes between different social groups, between displaced refugees and confined individuals, as well as in less mobile populations for example. The ability, however, to cultivate a sustained health-promoting microbiome may be difficult in certain marginalized populations, and yet, could be said to be a hidden foundational human right. This theme includes important research questions in the realms of sociocultural interactions in relation to the microbiome.

### 1. How do the microbiomes of displaced or migratory refugee populations compare to less mobile populations?

The risk of chronic inflammatory and stress-related psychiatric conditions is increased in members of urban communities, immigrants, and in particular, those moving from a low- to a high-income country during infancy (6, 7). In addition, the prevalence of chronic inflammatory disorders increases further in second generation immigrants, suggesting that critical exposures modulating disease risk occur during pregnancy and infancy (6). Although the mechanisms underlying the increased risk of chronic inflammatory conditions in immigrants are not clearly understood, the microbiota potentially plays an important role. Studying microbiome differences as a starting point may be important to the health of these communities beyond mere scientific knowledge.

Geographic location and ethnicity of individuals are consistently noted as fundamental drivers of microbiome composition. Gut microbiome biomarkers such as the genus *Bacteroides* are associated with high-income countries (and a diet richer in protein and fat), while *Prevotella* bacteria are associated with rural and low-income locations (and a diet richer in plant-based polysaccharides and fiber). Long-term stability of gut, skin, and oral microbiomes have been observed in stable lifestyles, but alterations occur when adapting to a new environment, culture, and diet.

In 2020, 91.9 million individuals were identified as people of concern by the United Nations (e.g., refugees, asylum seekers, internationally displaced people, and stateless individuals). Refugee research is a nontrivial undertaking, involving interactions with individuals that may be vulnerable and/or have a history of trauma. Vangay et al. (8) investigated the microbiomes of immigrants and refugees migrating from Thailand to Minnesota in the United States. The researchers observed that participants had reduced gut microbiome diversity and reduced plant-degrading glycoside hydrolase enzymes, proportional to the time spent in the United States (6). For example, refugees from the Karen community (Indigenous to the Thailand-Burma border area and impacted by conflict with Burmese military attacks) were included and had decreased *Prevotella* and increased *Bacteroidetes* after just 6 to 9 months following arrival. The data from these studies could help shift the centrality of “microbial health” as a concept away from something only associated with white wealthy populations. However, given the high level of vulnerability in displaced communities, existing processes for protecting vulnerable research participants is currently insufficient. The potential for unethical extraction of data must be considered and mitigated.

Given the logistical challenges of conducting research in refugee camps, one potential comparison group that may be more accessible, though still potentially vulnerable, are homeless individuals. The U.S. Veteran Microbiome Project has begun research into the skin, gut, and oral microbiome of an unstably housed population. Initial results showed that the frequency of homelessness was associated with gut microbial composition (9), thereby prompting future work focused on potential relationships between unstable housing, the microbiome, and physical and mental health.

### 2. Are there lifetime microbial consequences of early life adversity?

Adverse early life conditions are strong predictors of heart, lung, and autoimmune diseases, cancer, mental health disorders, and premature death (10, 11). The causes of such early life adversity can range from physical exposures to toxins, including lead, pesticides, or diesel exhaust (12), to aspects of early social environments like psychological stress (11, 13) and complex combinations of socioeconomic factors (14). Individuals are often exposed to multiple sources of early adversity, which may lead to even more extreme health outcomes (14). For example, a study of nonhuman primates found that individuals who experienced increased early adversity died a decade sooner than those who experienced no adversity (15). Further, effects of early adversity may manifest across multiple generations (12): offspring of nonhuman primates who experienced early life adversity also had early deaths (16).

Because humans are long-lived animals, it has been difficult to conclusively link early life adversity to lifetime microbial consequences. For example, microbiome composition in infants is shaped by delivery mode (cesarean section versus vaginal birth), antibiotic use, and diet (formula versus breastfeeding; acute malnutrition) (17–19). These factors likewise have been linked to childhood obesity, asthma, and type 1 diabetes (19), but multiyear longitudinal studies are needed to show that such effects are microbially mediated. In nonhuman systems, experimental studies suggest that early life administration of probiotics can ameliorate adverse immune and mental health outcomes, providing evidence of a relationship between early adversity and health-related microbial consequences (20, 21).

Taken together, the evidence to date suggests several important directions for future research. Do patterns and processes of early life microbial community assembly differ based on socially constructed factors (e.g., race, income, gender), and if so, why? What defines a healthy early life human microbiome, and how is this influenced by social inequality? How does early life adversity affect microbial aging (how the microbiome changes as people get older), and can early life microbial interventions reset the healthy microbial aging process?

### 3. Given the benefits of microbial diversity, what are the effects of social isolation and institutional confinement on the health provision of microbiomes to people?

Confinement is a continuum of socially sanctioned isolation settings ranging from the most segregated (prisons) to the most normalized (separated educational settings for students with disabilities) and may be best understood as the school-prison nexus (22). In the most punitive and isolated confinement contexts (i.e., youth detention, segregated educational settings, and prisons), where youth or adults of color with and without identified disabilities are disproportionately represented (23–25), a microbial equity lens highlights two main individual and population-level problems: (i) removal from and lack of beneficial microbial communities (26) and (ii) the potential for overexposure to pathogenic microbes (27, 28). Policies that normalize social segregation, isolation, and institutionalized confinement are effectively also policies that impinge on individual microbiota and broader microbiome health. Nevertheless, the relationship between social and microbial inequity remains poorly understood, let alone considered in social policy making and scientific agenda setting.

Incarcerated people suffer from higher rates of conditions linked to microbiome health (29), and barriers exist to providing individual meal plans and activity programs (30), further creating microbiome disruptions. Historically and currently, inadequate institutional protections create higher rates of infection in incarcerated populations due to lack of hygiene sanitation infrastructure, inadequate diet, overcrowding, and stressful conditions (27, 28). First-hand accounts of inmates receiving none to little personal protective equipment and access to vaccination are among the issues raised during the coronavirus disease 2019 (COVID-19) pandemic (31). Further, much of the focus on protection utilizes coercive measures such as mandatory blood testing and subsequent isolation, making prevention efforts punitive rather than protective (32). A floundering individual microbiome prior to, during, and after incarceration might exacerbate existing psychosocial and behavioral health challenges, such as mood, psychological disorder, and disease (see question 10 [Q10]), which have direct implications on recidivism, rehabilitation, and reform/revolution of the carceral complex.

These specific, objective, biological/ecological problems undergird more complex issues with broad ramifications for social equity and environmental justice and open up questions for future research. Do public health measures intended to reduce exposure to pathogens also reduce exposure to beneficial microbial assemblages, and does this matter? What comprises microbial equity for confined populations (for example, dietary improvements and greenspaces)? Could a health-promoting microbiome be conceived of as a right? What are the effects of social isolation on microbiome composition and downstream health effects, especially in incarcerated populations?

## II. INDIGENOUS COMMUNITY HEALTH AND WELL-BEING

Indigenous languages are fundamentally connected to the land and the environment from which they are spoken (33). Furthermore, there is a recognition of a direct link between language and traditional knowledge as they relate to biodiversity (34). Cognitive neuroscience has investigated culture as a phenomenon that shapes and influences various cognitive processes; behavior, schema formation, and memory (35–39). In this manner, Indigenous cultural practices influence how Indigenous Peoples form mental schemas related to nature, perceive nature, and remember and pass on practices related to nature. Understanding the relationship between Indigenous practices, cultural erasure, and Indigenous engagement in relation to the microbiome and its impact on Indigenous community health is covered in this theme. Indeed, the majority of human microbiome research to date has “focused overwhelmingly on populations predominantly of European descent, and typically those that surround large academic centres” (40). There has, however, been increasing interest in studying the microbiome of Indigenous Peoples (41–43).

### 4. What is the relationship between speaking an Indigenous language and practicing culture to microbiome integrity?

Indigenous Peoples languages around the world are fundamentally connected to the land and the environment from which they are spoken (33). Furthermore, there is a recognition of a direct link between language and traditional knowledge as they relate to biodiversity (34). Indigenous traditional knowledge as it relates to the environment is deeply embedded within Indigenous “names, oral traditions and taxonomies,” which can be lost when a community switches to another spoken language (34). With linguistic and biological diversity being functionally connected (44), a loss of Indigenous speakers and cultural-knowledge keepers has been shown in some regions to have a direct and negative impact on local biodiversity (45) with potential impacts on the microbiome. Yet, Indigenous knowledge has been recognized to be an important facet in curbing the loss of Indigenous languages and that of biodiversity (46).

“The microbial microcosm is a compelling narrative that situates our human biome in the biome of the planet, and in doing so, provides a common language to bridge efforts across and between movements, humans, and our natural environments” (47).

To date, there has been little to no examination of the relationship between speaking an Indigenous language and the microbial health of an ecosystem. The understanding of “co-benefits” has been clearly elucidated with protection of ecosystems and retention of Indigenous languages going hand in hand (34, 44–46). With the foundational elements of Indigenous environmental stewardship practices being rooted in Indigenous languages (48), more attention needs to be paid to how the microbial integrity of an ecosystem fits into this equation from a relational interspecies perspective. Furthermore, understanding how Indigenous languages and cultural preservation relate to the human microbiome may support Indigenous community priorities around revitalization efforts. Moreover, geospatial and metagenomic data may be used to support community-led studies between Indigenous health and Indigenous land conservation practices, which are strengthened through the continuation and application of Indigenous languages. Additionally, given the limited number of microbiology researchers whom identify as Indigenous, there is a substantial need to increase the accessibility of the microbiology field for Indigenous Peoples, while also eliminating structural barriers for funding and participation. Moreover, Indigenous sovereignty over any research process and data collected must be honored.

### 5. What is the effect of cultural genocide on the microbiome?

The Land and spiritual view many Indigenous Peoples embody directly influences the expression of culture (49, 50). Creating a reciprocal relationship, the Land informs culture, and culture informs Land-based practices. This mutualistic relationship plays a role in shaping how various Indigenous Peoples relate to nature; conservation, food production, land, air, water protection, care, and cultivation (51, 52). The Khasi Peoples of northeastern India and Bangladesh believe *time* creates strength in nature. This culturally driven perception instructs how the Khasi Peoples relate and care for biodiversity (53). Additionally, Robin Wall Kimmerer has played a key role in creating a framework for reciprocity with nature through using traditional ecological knowledge, which is based on her Peoples’ cultural and spiritual understandings of land (54).

These examples illustrate that Indigenous cultures give instruction on how to sustain biodiversity, which can promote health via the microbiome (55). If Indigenous cultures play a role in ecosystem health, conversely, cultural genocide could have an adverse effect. Insufficient land rights play a considerable role in cultural genocide as it removes Indigenous Peoples from their lands and communities (43–45). Once the removal happens, the cultural mechanisms that allow knowledge, ideas, and narratives to be passed on are destroyed, resulting in cultural genocide (56). Cultural genocide may therefore lead to changes in the microbiome through ecosystem disturbance and destruction. Insufficient land rights can also restrict the ability of Indigenous Peoples to hunt or otherwise gather food, restrict water access, and prevent use of lands for traditional migratory purposes.

Understanding more about how Indigenous Peoples’ practice of their own cultures impact the microbiome of the environment and the human host is a worthy research path. It is important to note, however, that many Indigenous Nations may not want to share all aspects of their culture as they are held within ceremony. Historical extractive actions and “biopiracy” by Western researchers have led to mistrust, and some Indigenous Peoples are reluctant to share their knowledge (57, 58). These perspectives need to be honored and reflected in any codeveloped and community-led research methodologies and community partnerships (46). Frameworks for enhancing ethical microbiome research with Indigenous communities are lacking; however, researchers may wish to draw upon similar established frameworks in genomics research (e.g., reference 59).

### 6. How do research institutions learn to better engage with Indigenous and other marginalized communities in microbiome research?

Extractive practices have unfortunately been a historical legacy in regard to research “on” or “for” Indigenous and other Minoritized Peoples as opposed to “by” or “with” (60), including in the area of microbiome research (61). Although some microbiome research has sought to address community concerns (62, 63), the vast majority has been carried out simply to “gain a better understanding of fundamental aspects of the human microbiome through the examination of minority populations, providing little or no direct benefit to the study population” (40). As microbiome research grows, including in the area of therapeutic interventions, concerns have been raised that the continued exclusion and lack of participation of minority communities in microbiome research will further exacerbate existing health disparities (63).

Community engagement designs that are responsive to Indigenous and Minoritized Peoples’ needs and concerns have greater potential to be leveraged into decreasing the research disparity gap. This means ensuring communities lead the way as experts in identifying existing processes, knowledge (past and present), as well as the questions communities may want to research. Supporting community-led initiatives will also help to prioritize and recognize the importance of community and data-level sovereignty (64), the importance of traditional knowledge, and ensure benefit-sharing processes are defined and implemented (65). Researchers also need to be mindful of the importance of strengths-based approaches to research in community-led partnerships as opposed to focusing only on deficit-based narratives and research models that risk further divides with communities (66). Collectively, these points drive home the repeated importance that microbiome research agendas in Indigenous and Minoritized communities should be “community-led” and “community driven” with supportive allyship present. Lessons may be learned in this respect from the developing literature in other synergistic areas such as Indigenous bioethics and Indigenous genomics (67–69).

## III. HUMANS, URBAN ECOSYSTEMS, AND ENVIRONMENTAL PROCESSES

Considerable work has linked climate change, pollution, and ecosystem degradation to reduced human health (65–72). Nonetheless, the roles these factors play in modifying microbial ecosystems and how they drive microbial inequality is relatively underexplored. This theme includes questions that ask how pollution, climate change, and ecosystem degradation affect the human and environmental microbiome, and how this may impact human health and social equity. We also explore how design and restoration of environmental microbiomes could be used in urban habitats to ensure equal benefits across society.

### 7. How does pollution affect the human microbiome, and are the impacts unequally distributed?

Pollution takes several forms, each with undesirable effects on human health and ecosystem integrity. Examples of chemical pollution include polychlorinated biphenyls in soils and airborne particulate matter. Exposure to these chemicals has been linked to immune dysfunction (61) and respiratory diseases (62). Several studies suggest chemical pollution can adversely affect the human microbiome. For example, xenobiotic-derived metabolites promote gut inflammation (63) and lead to changes in metabolism, immunity, and neurological function (64, 65). Ozone is associated with lower gut microbial diversity (66), and traffic-related pollution may negatively impact metabolic health via the microbiome (67). Noise and light pollution have increased to alarming levels across the world (68–70). Studies demonstrate deleterious impacts of noise on the mouse gut microbiome (72) with implications for host inflammation and associated diseases (73). One study showed that prolonged artificial light exposure can alter gut microbiota and promote nonalcoholic fatty liver disease (74). However, there is a clear deficit in studies exploring the effects of pollution on the human microbiome.

Social disparity in exposure to pollution is well documented. For example, people living in areas of higher deprivation are more likely to be exposed to poor air quality (75, 76). Therefore, the impacts of exposure are unequally distributed across social groups. The ultimate goal should be to reduce pollution and associated impacts to the lowest possible levels. This can be fulfilled through transdisciplinary solutions that promote equitable access to health-promoting environments (e.g., safe, biodiverse greenspaces), improving housing conditions, and policy changes that enforce improved monitoring and regulation of pollutants while subsidizing alternative sustainable sources.

Researchers should draw upon the aforementioned nonhuman animal-based studies for inspiration to investigate how chemical, noise, and light pollution may affect the human microbiome. Additional epidemiological studies investigating the impacts of pollution on the microbiome and human health could help to stimulate much-needed policy changes.

### 8. How will climate change and related ecosystem degradation affect the environmental microbiome, and what will the impacts be on health?

Ecological impacts of climate change include loss of ecosystem functions (e.g., carbon sequestration, climate regulation, ocean productivity, and crop yields), changing species distributions, and the emergence of new pests and diseases. These impacts can occur directly through context-specific climatic change (i.e., changes to precipitation, temperature, extreme weather events) or indirectly through altered delivery of ecosystem services (72). Ecosystem services are the myriad ways that people benefit from nature, including provisioning (food and fuel, energy, materials, medicines), regulating (habitat creation and maintenance, air quality, water quality), and cultural services (supporting identities and physical and psychological experiences) (72). Many of these ecosystem services have a microbial component where environmental conditions strongly control their distribution and activity. For instance, microorganisms in soils play a role in air quality and climate regulation, erosion control, water and waste purification, plant productivity, and disease control (73). Thus, as soil communities are altered through climate change, we should expect changes in the delivery of ecosystem services with implications for human health (77, 78). This is true for nonsoil environments as well, including host-associated microbiomes such as gut ecosystems (79).

Microorganisms play a key role in the human health outcomes currently experienced or expected to arise from climate change, including temperature- or air quality-related illnesses, vector-borne diseases, water-related illness, food safety and nutrition, and mental health and well-being (71). The vulnerability of groups of people to these health risks are a combination of their sensitivity, exposure, and capacity to respond (71). Socioeconomic status may affect which groups of people experience a higher burden of climate change health risk by influencing these risk factors. There are many pressing research avenues at the nexus of climate change, microbial ecology, ecosystems, and human health, including the following: (i) understanding the causal mechanisms between vulnerability factors for health risk and microbial functions, (ii) developing valid indicators for health risks and early warning systems, (iii) weighing the effectiveness of various measures that are designed to enhance resilience and reduce health impacts, (iv) uncovering the mechanisms underlying the relationships between ecosystem services and human health (78), and (v) predictive modeling of microbe distributions and functions and human health impacts (74).

### 9. How do we define microbial inequality in urban environments, and can we better identify, map, and characterize these zones?

The answer to this question ultimately lies at the intersection between microbial biogeography and the social, economic, and physical underpinnings of environmental justice. Environmental justice encompasses environmental “goods,” such as urban greenspace, as well as “bads,” such as air pollution. For humans, exposure to pathogens is considered “bad,” while exposure to diverse environmental microorganisms, which may foster healthy immune development, is considered “good” and has been proposed as a type of ecosystem service (80). To date, most research on geospatial relationships between health and microbial exposures has focused on microbial “bads” (81), which are often linked to a point or area source, such as waste treatment facilities. Urban slums can also provide breeding grounds for transmission of pathogens, due to overcrowding, poor housing quality, inadequate sanitation, and lack of access to clean water (75). On the other hand, urban sprawl is also leading to increased risk of vector-borne zoonotic diseases (76). Recently, spatial variation of microbial “goods” and their linkages with urban design have begun to receive greater attention (see, for example, references 80 and 82 to 91).

In our consideration of beneficial microbial exposures as a spatially distributed ecosystem service (80), we must first understand the “needs of the population” for microbial exposures and the degree to which microbial geographic distributions are influenced by human decisions and processes. Only then can we begin to operationalize policies and systems to promote distributional justice of microorganisms. Assuming that we could characterize certain microbial exposures as “goods” or “bads,” one promising approach for evaluating the equity of their spatial distribution is to use equally distributed equivalents (92). Benefits of using an equally distributed equivalent approach to mapping microbial exposures are: (i) absolute rather than relative abundance values are used; (ii) ability to objectively quantify exposure distribution to support decision-making; and (iii) “good” and “bad” microbial exposures are consistently and accurately represented.

Unfortunately, defining how much and which types of microbial exposures may provide health benefits to different human populations is currently an insurmountable challenge. No microorganisms are inherently “good” or “bad,” rather their pathogenic or salutogenic potential is determined by context and may change over time or with ecological disruptions (e.g., antibiotic use). Racial, ethnic, cultural, religious, gender, socioeconomic, and other demographic factors may also influence the relationship between microbial exposures and individual well-being.

### 10. How can safe and health-promoting environmental microbiomes be designed and/or restored in urban habitats to ensure equal benefits across society?

It has been argued that microbiota should be considered ecological determinants of health, given their critical role in human health and the interrelated systems we depend upon for survival (93). Access to plant-associated microbial diversity seems to offer health benefits to humans through microbial colonization (94–96). It is necessary to understand microbiomes in a socially just framework to ensure equal access to these benefits. The structure and function of microbial communities are known to covary with the urban landscape in the air (97–100), soil (101–103), and built environment (104–106). Urban microbial diversity is patchy, and greenspaces are hot spots for plant-associated microbes. Likewise, greenspaces are unevenly distributed through cities and may lack the diversity offered by access to nonurban nature (107). Use of greenspaces (and subsequent access to microbial benefits) is not determined solely by proximity. It may depend on human residential stability (107) and the physical/emotional safety of the spaces (108).

By altering the structure of the urban environment to increase greenspace outdoors (109) and greenness indoors, it may be possible to promote safe and healthy microbial access in cities for all residents. The use of small land parcels such as vacant lots could be used for microbial restoration interventions to provide a more equitable coverage of biodiverse habitats across urban landscapes (110, 111). Likewise, greater incorporation of green infrastructure (112) into the built environment, such as offices and shops, will offer more equitable access to microbial benefits. Greater awareness and education of microbial ecosystem services will help urban planners and policymakers design safe and health-promoting microbiomes. Dissemination of microbial information and access can be offered to stakeholders and decision-makers through a series of lectures, workshops, and conferences. In general, providing this information in an intelligible (i.e., nonscientific) way is key, as the majority of the conversation to date has been rooted within academia.

## IV. HUMAN PSYCHOLOGY AND MENTAL HEALTH

### 11. Is there a relationship between the microbiome and mental health, and are vulnerable populations differentially affected?

In 2004, the “Old Friends” hypothesis was introduced (113). This hypothesis proposed that a failure of immunoregulation and associated health problems (e.g., mental health conditions) in modern Western environments (83), indicated by a balanced expansion of effector T-cell populations and regulatory T cells (Treg), is due to reduced exposures to microorganisms with which humans coevolved, including: (i) pathogens associated with the “old infections” that were present throughout life in evolving human hunter-gatherer populations (114); (ii) the commensal microbiota, which have been altered by the modern Western lifestyle, including a diet that is frequently low in microbiota-accessible carbohydrates (115–117); and (iii) organisms from the natural environment with which humans were in daily contact with (and, consequently, had to be tolerated by the immune system) (83). More recently, the observation that two major socioecological trends (i.e., the loss of biodiversity, and increasing incidence of inflammatory diseases) are interdependent led to the biodiversity hypothesis (67, 99, 100). This states that people’s reduced contact with nature and environmental biodiversity has altered the human commensal microbiota’s capacity to induce immunoregulation and to prevent inappropriate inflammation as well as associated negative health outcomes (7, 89, 118).

In light of evidence which supports the hypothesis that lack of exposure to diverse microbial environments can lead to chronic low-grade inflammation and increased risk of stress-related psychiatric disorders, recent studies point toward potential interventions to restore immunoregulation (e.g., probiotics) (119). Moreover, it is clear that living in close proximity to greenspaces reduces overall mortality, cardiovascular disease, and depressive symptoms and increases well-being (120–124). Initiatives such as the Create Outdoor Equity Grant Program in Colorado, designed to increase access and opportunity for underserved youth and their families to experience greenspaces, state parks, public lands, and other outdoor areas, have promise to both increase microbial exposure and increase social equity (125). Perhaps now more than ever, initiatives that focus on access to greenspace and diverse and healthy diets have potential to increase not only physical health but also mental health, on local and global scales. Knowledge gaps include guidelines on best practices for maximizing nature exposure and whole dietary and probiotic interventions with potential for enhancing immunoregulation, limiting inappropriate inflammation, and reducing the risk of mental and physical health conditions.

Might centering microbial equity as a facet of purported “rehabilitation” advance the creation of institutions that, as Angela Y. Davis presciently said nearly 2 decades ago, could “lay claim to the space now occupied by prison [in order to] eventually start to crowd out the prison so that it would inhabit increasingly smaller areas of our social and psychic landscape” (reference 253, pages 107 and 108).

## V. THE MICROBIOME AND INFECTIOUS DISEASES

Infectious diseases affect human microbiomes and may exacerbate socioeconomic disparities. Indeed, some communities are disproportionately exposed to infectious disease agents due to anthropogenic environmental change. Moreover, populations without the resources to recruit a beneficial microbiome require interventions to prevent and treat associated infections. This theme discusses these phenomena in addition to asking the question of what lessons have been learned from pandemics, in particular, the implications of remote working for equity, and protection from infection.

### 12. How might infectious diseases affect human microbiomes, and does socioeconomic status influence this relationship?

The human microbiome is described as having approximately 3.3 million nonredundant microbial genes, which is about 150 times more than the human genome (126). Despite this, the associations between microbiomes, infectious diseases, and socioeconomic status (SES), are relatively unexplored. In 2015, Logan argued that in *“*western industrial nations a “disparity of microbiota” might be expected among the socioeconomically disadvantaged, those whom face more profound environmental forces*”* (127). This was corroborated in a recent study which examined the association between gut microbiota and social factors in twin cohorts. Study participants that experienced health disparities reportedly had lower SES and education levels than those that did not (128). Participants with lower SES reportedly had lower alpha diversity in gut microbiota, which can influence health factors such as metabolism, gene regulation, and host immune responses (128–130). In other studies, lower neighborhood SES has been associated with reduced diversity of colonic (131) and salivary microbiota (132), and family SES is associated with the gut microbiome in infants and children (133, 134).

Historically, infectious disease research has focused on pathogens alone; however, more is being learned about the host-pathogen relationship and its effects on disease establishment and long-term health effects (135). Outcomes of infectious disease depend greatly on the microbial inhabitants of an individual (136). In 2007, the Human Microbiome Project was launched by the National Institutes of Health and started the developing field of microbiome research (137). Since that time, countless discoveries have been made regarding the complexity and interconnectedness of microbes associated with humans. For example, a person’s gut microbiome influences other body processes like metabolism, disease, and neurological processing (138). A stable and diverse gut microbiome will also aid in the regulation of homeostasis via, for example, cell signaling, and the production of health-promoting metabolites (e.g., the short-chain fatty acids butyrate, acetate, and propionate) (138). Many other body sites have a microbiome, and they are relatively underexplored. The skin, lung, vaginal, and oral microbiomes for example, are all important for human health. Their role in regulating diseases requires additional research.

The reciprocal relationship between one’s microbiome and immune system can influence the immune response, thus potentially affecting a person’s susceptibility to infectious diseases (136). One’s physiology is a result of the metagenome, or the individual’s genes combined with microbial genes (139). The countless sets of microbial genes affect the expression of our own human genes (139). The complex relationships between host and microbiome are largely unknown. One of the most widely studied microbiomes is the metabolic network of the human gut. Microbes found in the human gut can downregulate or upregulate genes that result in metabolic disorders, gastrointestinal disorders, and psychological disorders (140). These conditions can be important risk factors for infectious disease severity (141).

In communities that experience deprivation, an individual’s metagenome will most certainly be affected. It is in these communities that greater numbers of microbiome-influenced diseases are reported (6, 128, 142–144). Vulnerable populations and the trajectory of immune responses as a result of social inequities need to be examined more closely. This is one pathway to establishing a more equitable society.

### 13. In populations without the resources to recruit a beneficial microbiome, how can we incorporate microbiome interventions into the prevention and treatment of infections?

Certain microbial community members are of greater importance for the host and for supporting microbial stability (145). Collectively, a stable microbial community can make the human host less susceptible to infection (146). This decreased susceptibility has been demonstrated through microbial competition for nutrients, antimicrobials, biofilm formation, and physical exclusion on host cell surfaces, disruption of other microorganisms’ biofilms, and other mechanisms (reviewed in references 147 and 148). Commensal microorganisms can help prevent infections by improving host immune function; for example, by increasing gene expression for mucin production, and stimulating protein synthesis to enforce tight-cell junctions between gut epithelial cells (reviewed in references 147 and 148).

Using microbial therapeutics (e.g., probiotics) to treat active infections may be effective, and their use is becoming more common in medical practice in conjunction with, or as a replacement for, antibiotics (149, 150). Yet, potential treatment with probiotics requires the time, money, availability of relevant strains, and interest in identifying the infection and a suitable probiotic treatment. For anyone without access to affordable, culturally safe, and equitable health care services (151), obtaining personalized treatment resources may be extremely difficult. With prevention strategies likely being more effective than reactive postinfection strategies, examining the use of dietary-based beneficial microorganisms remains a promising cosolution (152).

Poorly functioning microbiomes compound over time and over generations (115). Long-term solutions are required to ensure access to fresh, high-fiber, and nutritious foods over a lifetime—long enough for stable microbial communities to promote a healthier host (153, 154). It is not a lack of scientific knowledge which hinders the use of diet to recover beneficial gut microbiomes, which engenders the elegantly simple question: *so why don’t we*? Establishing or recovering functional gut microbiomes involves being able to acquire and retain microorganisms, which necessitates commitments to diet or lifestyle changes, and importantly, the means and resources to engage in these changes. Simply stated: you cannot eat more vegetables if you cannot acquire them. For this, we need to stop thinking about strategies for “microbial-based intervention” and instead focus on social programs which support “microbial-promoting lifestyles”. Future research efforts should focus on identifying barriers to equitable implementation from the personal to community scale. For example, lack of dental care access and poor oral health can impede fiber intake (155). Again, it is important to acknowledge that other body sites have microbiomes that are relatively underexplored and require further research to determine their potential roles in the prevention and treatment of infections.

### 14. Are some communities disproportionately exposed to infectious disease agents due to anthropogenic change, such as habitat destruction, human population migration, and climate change?

Anthropogenic land use change and climate change are important drivers of infectious disease outbreaks (156–160). These two drivers result in increased human contact with wildlife hosts of infectious disease via biodiversity loss, habitat fragmentation, range shifts, and migration (156, 161, 162). The impacts of land use and climate change (including disease exposure) are not equally distributed across landscapes or human societies (72, 163, 164). For instance, communities with livelihoods dependent on land conversion, living close to degraded land, or with a changing climate may have greater exposure to infectious disease agents (72, 165). Those involved in the wildlife trade also face higher exposure (72). Furthermore, land degradation is an important driver for human migration (166), which is associated with poor health outcomes due to a lack of pre- and postarrival health care. This could potentially lead to infectious disease outbreaks (164, 167) and substantially affect vulnerable communities (168).

In particular, women and Indigenous communities may be among the groups most at risk of infection (72). Globally, 70% of the world’s poor are women, and 70% of the world’s health care workers are women (169). Moreover, the colonization of Indigenous land and Peoples continues to result in health inequalities and a lack of culturally appropriate resources, contributing to a higher risk of infectious disease (72, 170).

Recent UN reports on infectious disease indicate that most governments worldwide are concerned about social equity and exposure to emerging diseases (171). We echo their call for collaborative land protection and rehabilitation that is sensitive to local context (172) and Indigenous knowledge (173, 174). For instance, Indigenous Peoples’ land sovereignty can provide an opportunity to decolonize land practices (175) and strengthen the resilience of communities especially when resource distribution is disrupted by events like a pandemic (171). In addition to basic research on land use change (176), climate change (177, 178), and emerging diseases (179), we need a better understanding of the degree to which restoration and sustainable land management reduce disease exposure. Outcomes of this research and benefits from the health system need to be shared equally (72) and incorporated into just, diverse, equitable, and inclusive policy.

### 15. What lessons have we learned about remote working during pandemics, and what are the implications for equity and protection from infection?

The impact of natural disasters and financial recessions on populations is determined by occupation, education, income, housing situation, structural bias, and discrimination (180–182). COVID-19 has made these inequities more apparent. Individuals with low SES or minority status experience disproportionately higher COVID-19 infections, hospitalizations, exposures, financial hardship, and deaths (183–187). Preliminary research connects COVID-19 with the microbiome. For example, altered gut microbial metabolites could mediate COVID-19 risk factors, and gut microbiome dysbiosis may play a crucial role in the poor outcomes of COVID-19 in elderly, diabetic, and hypertensive patients (188, 189). Infectious disease exposure in individuals with lower SES and minorities is not exclusive to COVID-19 (190). Studies have repeatedly demonstrated the disproportionate impact of tuberculosis (191), sexually transmitted diseases such as HIV/AIDS (192), and respiratory diseases (193) on these groups as well.

A key venue of disease exposure is in the workplace. The requirement for continued income may outweigh potential risks of exposure (194) and can lead to delaying doctor visits until absolutely necessary (195, 196), thus increasing the likelihood of severe outcomes (176, 197). Employers can take evidence-driven steps to promote safe and equitable working conditions. Mandated paid sick leave when ill can decrease transmission while reducing concerns over diminished wages (198, 199). Providing guidance in selecting care can also result in increased access and utilization of health care services (200, 201). Quarantine is understandably not an option for everyone. In situations where employees are not able to perform duties from home (e.g., for “key workers” such as medical staff or manufacturers of essential goods), increased pay should be considered. However, employers should strive to create a flexible and inclusive work culture and environment, and where possible, include the option of remote working (202, 203).

There is already a broad understanding of the steps that can be taken to decrease infectious exposures, and individual organizations should investigate their own policies and practices to limit the risks of infectious disease exposures to their employees or customers. However, employees working remotely, especially from home, may not have the resources or agency (i.e., renters) to improve the quality of their built environment. Research into simple and affordable strategies to improve building air, sound, and light quality can improve occupant well-being. Research into the feasibility of improving old building stock can identify where to focus renovation efforts to maximize health interventions, for example, public workplaces versus apartment complexes.

## VI. HUMAN HEALTH AND FOOD SECURITY

### 16. How do we reorganize food systems to improve food availability, security, supply chain operations, and economic viability of agriculture, while promoting access to fresh foods to support a diverse microbiome?

Today’s global food system and its corollary modes of production, processing, transportation, and distribution are a product of centuries of colonialism, forced migration and enslavement, genocide, extraction of natural resources, and exploitation of human labor (204–206). Human health and well-being garner minimal concern within the industrialization of food systems as capital and private interests dictate the parameters of production and distribution (207). Structural inequalities embedded within food systems constrain possibilities for humans—and the microbial communities inhabiting them—to flourish (208). Indeed, commercial tactics in minority and deprived communities are a fundamental part of structural poverty and structural racism (209). Arguably, the entire retail environment in disadvantaged communities (and the larger neighborhood environment) is commercially engineered to promote the consumption of unhealthy, ultraprocessed foods by default, foods that are detrimental to the gut microbial ecosystem (127, 210).

The global industrial food system is a leading cause of climate change, environmental degradation, and biodiversity loss. Even those of economic means may struggle to nourish a diverse microbiome – or alternatively, buy their way *out* of an imbalanced, unfavorable microbial environment (211, 212).

Therefore, instead of “food security,” we adopt a paradigm of “food sovereignty,” which offers a more comprehensive path toward solving the intersecting crises of human health and the environment (213). “Food sovereignty” addresses structural barriers and discrimination that hamper access to farming and other land-based livelihoods among Black, Indigenous, and other communities of color (214, 215). Additionally, it calls for systems of production and supply chain operations to shift away from fossil fuels and instead adopt green energy and regenerative practices that restore biodiversity to soils and ensure clean air and water to support surrounding human and nonhuman populations; agroecology could help remedy these multidimensional crises (216).

More research is needed to examine how to adopt a human rights-based approach to food frameworks while also dismantling the stigmas around welfare assistance and the commercial tactics that drive structural poverty and promote the nefarious cycle of ultraprocessed food consumption (210). In terms of microbes, research must examine the effects of industrial diets such as the “Standard American Diet” on gut microbiomes, and of other variables that shape microbial communities (212, 217). Comparative research is also needed to examine the structural constraints faced by people with less diverse or favorable microbiomes versus those with more favorable ones (2). Finally, it is imperative that research and policy both protect food system workers—providing them with living wages, health insurance, paid sick leave, and other benefits—*and* uphold the dignity of people in efforts to ensure equitable distribution of food resources and to abolish “food apartheid” (Karen Washington quoted in reference 205).

## VII. MICROBIOME-RELATED PLANNING, POLICY, AND OUTREACH

Microbiome-related planning, policy, and outreach are imperative to the successful implementation of interventions that seek to reduce social inequities in the microbial realm. However, before seeking new solutions, there may be existing initiatives that already promote these interventions, or that can be transferred to this area. Reforms are needed in health care to address our understanding of chronic stress on the microbiome. Moreover, how to improve the translation of microbiome research findings to address social equity requires deep consideration. This theme contributes to these important policy-related phenomena, to frame the microbiome as a facet of social equity and stimulate much-needed policy discourse.

### 17. Before seeking new solutions, what are the existing community initiatives and grassroots movements that push for impacts on microbial, social, and biological equity?

We are unaware of community initiatives to date which explicitly work toward microbial equity in any context. However, there are many community organizations and grassroots movements which effectively—and perhaps inadvertently—promote microbial exposure equity by providing resources to obtain beneficial microbial exposures (e.g., convivial activities in nature), protecting against harmful microbial exposures (e.g., paid sick leave, personal protective equipment, or hygiene facilities), and supporting health and productivity. A framework for this is presented in Fig. 1.

Providing resources to obtain beneficial microbial exposures may include food, water, environmental spaces, or housing security. Supporting a healthy and productive gut microbiome is a side effect of programs which support local food production systems, integrate local food production into education systems to provide healthy meals, improve availability of fresh foods, create community gardens, and incentivize business development to reduce food deserts. Similarly, creating equitably located and designed public greenspaces in urban areas can promote beneficial exposures to environmental microbiota. Flourishing urban green infrastructure (including the microbial components) can reduce wastewater overflow, which could contaminate freshwater sources, and increase shade to cool neighborhoods. This could potentially reduce heat stress and the associated “leaky gut” or microbial dysbiosis (218–220).

Initiatives that support housing renovation, particularly after natural disasters that lead to microbial degradation of infrastructure, can protect people from harmful exposures (221, 222). So, too, can organizations which promote paid sick leave, thus reducing the spread of infectious diseases (185, 199). Additionally, medical and dental preventative care across industries and extended to part-time, contract, and temporary employees, could also be valuable in preventing harmful microbial exposures.

Finally, community initiatives that support health and productivity most often focus on reducing stress and improving quality of life. However, these are posited to beneficially impact host-microbe interactions due to the reduction of stress-related inflammation (223, 224). Improving safety and accessibility of public transportation, reducing harm caused by institutional racism or sexism, improving indoor and outdoor air quality, and other solutions can reduce microbial exposure inequity by removing those problems that disrupt human microbiomes and cause further harm (219, 225–227).

### 18. What types of reforms are needed in health care and policy, in order to address our understanding of chronic, socially mediated stress on the microbiome?

The cultural context that creates and supports the cognitive architecture of the people making health care decisions and policy must be reviewed. Racialized white Western culture has a long history of supremacy; over people, land, and thought. Knowledge supremacy is defined here as the phenomenon of a homogeneous, discriminating, and dominating knowledge pool (211). It can corral society’s imagination, and thus contributes to a hegemonic ruling class over all aspects of our society (228), which can include health care frameworks and policy.

Arguably, current health care practice and policy fail to include sufficient ecological knowledge (229). This centers the responsibility for health on the individual. For example, the “five-a-day” campaigns (referring to the consumption of fruit and vegetables), “sugar tax” (the taxation of any product with a high sugar and/or fat content), or the calls for more physical activity, all define health within the context of individual choices. These approaches ignore the requirement of policies that protect people and communities from environmental pollutants, health-demoting habitats (e.g., lacking in biodiversity, safety, accessible recreational spaces), and poverty, which weigh heavily on health outcomes as they cause systemic changes to both human and environmental microbiomes (230). The effects of environmental stress and insufficient microbial exposures likely impact human health (e.g., via dysregulation of the immune system) (231). Emerging research highlights how stress from environmental pollutants or psychosocial pathways can affect the internal human microbiome through the gut-hypothalamus-pituitary axis (232), which can potentially contribute to an array of adverse health outcomes. Additionally, because health is rarely framed from an ecological perspective, current health care policies are insufficiently aligned with paradigms that support the restoration of biodiversity, including the environmental microbiome.

Integrating knowledge of the microbiome at a systemic level will likely create new opportunities to optimize health care practices and policies that support equitable health outcomes. Transdisciplinary collaborations are required to address social (including racial) health disparities in microbiome and health research (233).

### 19. How do we improve the translation of microbiome research findings to address social equity?

The translation of microbiome research into practice is already under way by the biotechnology sector. Examples range from popular communication (TED talks), the commercialization of self-directed precision medicine via direct-to-consumer sequencing (Viome), and the advertising and myth making around the microbiome as a cure-all (234). All of the above continue to concentrate power and capacity in high-income countries while extracting value from marginalized communities. Acknowledging the shared path with businesses, policy, and society, the research community can directly center equity as a key value driving the translation of research into practice.

The translation of microbiome knowledge and research into technologies, policies, and programs that ensure equitable distribution of benefits is determined by who gets to shape them and on what grounds (235, 236). We suggest a deliberately inclusive and proactive approach that recognizes the existing biases of the research community on lines of race, class, gender, sexuality, and disability (237) and the limitations of using unmarked stakeholder types. Furthermore, deliberate geographical inclusion is needed to address the representation and resource imbalance favoring both researchers and researched populations in the current G7 countries; the United States, Canada, the United Kingdom, France, Germany, Italy, Japan, and the European Union (238).

To address the pervasive lack of trust in science-led policy as revealed by the COVID-19 pandemic (239, 240) and build trust, agency and capacity across global communities, it is key to recognize that stakeholder engagement is equitable only when it genuinely gives power to a wide range of social groups (241) and does not mine lived experiences for upstream value in the scientific and business communities (242). As we move toward formulating actionable points for diverse fields, further participatory research is needed to ensure blind spots (e.g., cognitive biases) are recognized and addressed.

Microbiome research also needs to reckon with a fear of “germs” that is present in the public imagination and heightened by the COVID-19 pandemic. Many artists and designers have been working on projects that build awareness of the importance of the microbiome beyond pathogens: *Host* by Baum and Leahy and Richard Beckett (243), *With Microbes* by Ioana Man (244), and *Subculture: Microbial Metrics And The Multi-species City* by Kevin Slavin, Elizabeth Hénaff, and The Living (245). Additionally, and to diversify the audience of this kind of work, it is important to recognize that the interdisciplinary approaches to understanding the microbiome are part of people’s cultures and ancient practices across the globe (246, 247). It is also important to expand the creative imagination around creative outputs to include them. Design can push for equity by allowing scrutiny of what the implementation of new research findings could look like in different communities and situations (248). For instance, the design component of Microbiome Inspired Green Infrastructure (249) opens up what our cities might look like if we were to design green infrastructure with the microbiome in mind and starts to solicit feedback from diverse stakeholders. These forms of transdisciplinary collaborations can help us develop a common language and open forums needed to solicit diverse perspectives required to distribute the benefits of research findings in an equitable manner.

## DISCUSSION AND CONCLUSION

### 20. What is the socioecological and political potential of learning about social inequities in microbial exposure?

The questions posed in this paper raise acutely challenging questions for policy makers. The study of the health benefits of microbial exposure highlights perhaps the greatest challenge for policy makers—developing policy in the absence of complete information. This is familiar terrain for researchers in this field, where important discoveries have been made but much is yet to be resolved. In the first instance, making use of the particular skills needed to recognize and navigate blind spots and preconceptions will make a transformative difference to policy making. Codesign and delphi-type research exercises can help, but questions can be asked in terms of what more can be done to drive policy, and what is most effective. Amid increasing awareness of the importance of intersectionality in developing policy goals and translating scientific research into practice, “microbes and social equity” research is highly interdisciplinary, a facet that will be central to progress in this space.

The 20 important research questions in this paper are consistent with several of the UN sustainable development goals (SDGs), either directly or indirectly. For example, *Goal 3: Good Health and Well-being*, *Goal 10: Reduced Inequalities*, and *Goal 11: Sustainable Cities and Communities*. The review highlights that the microbiome is essential to favorable health and well-being (Goal 3); and there are likely significant inequities in microbial exposures (Goal 10); furthermore, functional microbial communities are essential to flourishing ecosystems, and therefore to healthy and sustainable human communities (Goal 11). Consequently, the knowledge gained from answering these 20 questions can make a vital contribution to supporting the progress of the SDGs. Moreover, the microbial exposure and social equity questions here directly overlap with the *One Health*, *Planetary Health*, and *EcoHealth* frameworks, and fit within the realms of antibiotic stewardship, and rural health initiatives. Therefore, integration within these established frameworks in practice will likely increase the effectiveness of strategies and reduce incommensurability between disciplines.

The thematic categories identified in this paper reinforce messages that arise from other horizon-scanning exercises, first among which is the importance of research-led policy making. For example, when modellers work collaboratively with policy makers (e.g., in Australia and New Zealand [250]), effective interventions can be designed. See Q9 for more details.

However, this need for research-led policy making in an environment where important data are missing reinforces the importance of recognizing cognitive biases and developing tools to make progress in the absence of complete information. We are becoming increasingly aware that negative impacts on human health and ecosystems are the result of cumulative and interconnected processes (251), and optimizing how we measure and value information is imperative to identifying critical pathways, decision-making, and design. This relies on embracing probability in decision-making: at present, focus tends to be on biology rather than psychology, and certainty rather than probability. It would be prudent to recognize the value of likelihood, possibility, and gradients rather than choices between alternative options.

International and national policy frameworks are increasingly refined and nuanced in addressing inequities. However, while scientific research can help address technical challenges, these developments will be successful only if they are translated into a language that is understood by nonexperts. Recent political experiences have highlighted the difficulties in reaching consensus or shared understandings of needs. In this way, embracing probability—not only as a means of weighing competing needs in policy discussions—but in wider political discourse with an electorate, could help identify and reach shared value systems. Research in this field is urgently needed to develop new tools that can help address cognitive biases and encourage the imagination that is required to identify and reach shared goals.

The health of an individual is something that can be assessed and quantified and has its own measures of resilience and functioning. In contrast, equity is relational and describes the condition of an individual relative to another in a community. Further research is needed so that policy makers can better articulate questions that manage this balance between the health of an individual and the basic human tendency of seeing our own situations in relation to others’. It is imperative that we address the nefarious commercial tactics in minority and deprived communities that drive structural poverty and racism. These tactics also drive the continuation of the vicious cycle that underscores disparities in health via the microbiome. This will not be an easy task as the same commercial pressures affect political will; however, promoting discourse on this topic is an important starting point.

It is also imperative that we advance equity and inclusion in microbiome research and training. Foxx et al. recently proposed ways to improve inclusion in microbiome science, advocating for resource expansion to enhance capacity (252). The authors urged mentors, collaborators, and decision-makers to commit to inclusive and accessible research to correct the inequities imposed by structural socioeconomic disparities involving wealth, class, and race. We echo these calls.
